# Identifying Key Hospital Service Quality Factors in Online Health Communities

**DOI:** 10.2196/jmir.3646

**Published:** 2015-04-07

**Authors:** Yuchul Jung, Cinyoung Hur, Dain Jung, Minki Kim

**Affiliations:** ^1^Korea Institute of Science and Technology InformationDaejeonRepublic Of Korea; ^2^Electronics and Telecommunications Research InstituteDaejeonRepublic Of Korea; ^3^Korea Advanced Institute of Science and TechnologyDepartment of Business and Technology ManagementDaejeonRepublic Of Korea; ^4^Korea Advanced Institute of Science and TechnologyCollege of BusinessSeoulRepublic Of Korea

**Keywords:** hospital service factors, online health communities, social media-based key quality factors for hospitals, recommendation type classification, quality factor analysis, healthcare policy

## Abstract

**Background:**

The volume of health-related user-created content, especially hospital-related questions and answers in online health communities, has rapidly increased. Patients and caregivers participate in online community activities to share their experiences, exchange information, and ask about recommended or discredited hospitals. However, there is little research on how to identify hospital service quality automatically from the online communities. In the past, in-depth analysis of hospitals has used random sampling surveys. However, such surveys are becoming impractical owing to the rapidly increasing volume of online data and the diverse analysis requirements of related stakeholders.

**Objective:**

As a solution for utilizing large-scale health-related information, we propose a novel approach to identify hospital service quality factors and overtime trends automatically from online health communities, especially hospital-related questions and answers.

**Methods:**

We defined social media–based key quality factors for hospitals. In addition, we developed text mining techniques to detect such factors that frequently occur in online health communities. After detecting these factors that represent qualitative aspects of hospitals, we applied a sentiment analysis to recognize the types of recommendations in messages posted within online health communities. Korea’s two biggest online portals were used to test the effectiveness of detection of social media–based key quality factors for hospitals.

**Results:**

To evaluate the proposed text mining techniques, we performed manual evaluations on the extraction and classification results, such as hospital name, service quality factors, and recommendation types using a random sample of messages (ie, 5.44% (9450/173,748) of the total messages). Service quality factor detection and hospital name extraction achieved average F1 scores of 91% and 78%, respectively. In terms of recommendation classification, performance (ie, precision) is 78% on average. Extraction and classification performance still has room for improvement, but the extraction results are applicable to more detailed analysis. Further analysis of the extracted information reveals that there are differences in the details of social media–based key quality factors for hospitals according to the regions in Korea, and the patterns of change seem to accurately reflect social events (eg, influenza epidemics).

**Conclusions:**

These findings could be used to provide timely information to caregivers, hospital officials, and medical officials for health care policies.

## Introduction

Patients and their caregivers increasingly use the Internet for health care information sharing and support [1]. In particular, they tend to obtain health information and share health experiences through social media services, such as Wikipedia, Facebook, online forums, and message boards [2]. Overall, social media apps have the potential to enhance health equity: interaction through social media can increase social support and feelings of connectedness and lead to a sense of empowerment in patients. Such interaction also increases information sharing, which in turn leads to a more patient-centric experience. For this reason, researchers studying health communication among patients have also focused on online user-generated data on health, for instance physician rating websites [3], blogs with health-related keywords [4], free-text comments about hospitals on websites [5], and message boards in online health communities [6].

Hospital quality is among the most interesting health information to patients, and many studies conducting hospital quality analysis have relied heavily on random sampling surveys [7,8]. However, the practicality of random sampling approaches is limited because they require significant human effort and are thus labor intensive and time consuming. An Internet-based survey is one alternative, but specially designed questionnaires are often limited to paid users. In addition, it becomes more difficult to apply traditional statistical approaches to health-related information owing to the tremendous volume of health-related information generated daily. Surveys regarding hospitals reflect users’ opinions on the hospital’s service quality and patient satisfaction, but social media can deliver the same results more readily.

Social media is a valuable resource when deciding which hospital to choose. For instance, messaging systems like Twitter and Facebook allow words and phrases to be searched using algorithms to find positive or negative sentiments [9]. Recently, a study [10] showed that a hospital’s Facebook page could be a quality indicator. They found that the number of “likes” on a hospital page was an important indicator not only of patient satisfaction, but also of quality of patient care. In particular, based on observation of 40 hospitals in New York, a correlation was found between Facebook “likes” and patient recommendations as well as declines in 30-day mortality rates (ie, for every percentage point drop in the 30-day morality rate, there were nearly 100 more Facebook “likes”). Although it can be difficult to generalize much from correlations, the more important point is that widely accessible social media can be a meaningful indicator of hospital quality.

To obtain useful information from online communities, several automatic approaches have recently been reported. Investigations on various social media such as blogs, bookmarks, communities, and forums have applied topic modeling [11]. Different topic modeling strategies were considered depending on the type of social media because each social media has different document types and metadata. For example, to find an online community expert, not only authorship but also the volume of social feedback, such as comments, likes, and shares are key factors for estimating expertise. Recent studies [12-14] have used quantitative summaries of user-generated content such as overall valence and volume of user review ratings to represent user opinions. In contrast, very few recent studies formally incorporate and test the influence of the textual content of user-generated reviews [15,16]. Researchers [11] have analyzed online user-generated reviews for the hotel industry (eg, TripAdvisor) to better measure hotel service quality and performance. They have attempted to create a more comprehensive view of online user-created content by considering both quantitative aspects and textual context from multidimensional perspectives by applying text classification and sentiment analysis techniques. Further, there is a hospital service quality version of sentiment analysis that uses Twitter data. For instance, Chou et al [17] found that the “Mayo Clinic” was rated as negative, but an examination of tweets mentioning the clinic showed a mix of sentiments. Some that were rated negative (78%) were associated with concern about someone in surgery or with having an illness rather than with care or quality.

Although various types of social media can be used to obtain health-related information, online health communities are the most pertinent for several reasons:

1. We can focus on social interaction between those who question and those who answer. In online health communities, patients and their caregivers can share their experiences and exchange hospital and medical information. In particular, emotional impressions and qualitative evaluations of hospitals offered by community members are crucial points for patients and caregivers when deciding which hospital to choose.
2. Convenience and anonymity are important reasons why mothers use the Internet [18]. They expect to find health-related and hospital-related information easily and quickly, and they are typically not embarrassed to ask questions of online health professionals or communicate with online members about their personal conditions and experiences [19].
3. Online health communities enable patients to take a more active role when choosing an appropriate hospital through the use of social support. They can consider multiple aspects such as “service”, “professionalism”, “process”, “environment”, “impression”, and “popularity”.

Therefore, we selected user-generated messages from online health communities for our experimental data.

We aimed to reveal the details of hospital reputation by not only conducting sentiment analysis but also analyzing multidimensional service quality factors inside users’ written messages. Our proposed approach does not only rely on the number of “likes” or “recommendations” but finds out which hospital service quality factors are really associated with the recommendations of online health communities. In summary, our contributions are threefold:

1. We define the social media–based hospital service quality factors model consisting of six service quality factors: “service”, “professionalism”, “process”, “environment”, “impression”, and “popularity”, based on observation of the content in online health communities.
2. We propose a novel approach to detect social media–based hospital service quality factors using text mining techniques. Our key aim in this task was to automatically identify these factors in online health communities effectively. To this end, we implemented a set of text mining modules to extract potential clues to those service quality factors and to classify them into two recommendation types. Detailed steps include focused crawling, preprocessing, dictionary-based hospital name extraction, filtering, detecting quality factors for hospitals, and recommendation classification.
3. We provide the analysis results that can be visualized as patterns of major behavior change factors (for hospitals that were negatively assessed by the general public) by analyzing the six main cities in Korea. The analysis shows the effectiveness of our proposed methods to reveal the potential of collective intelligence based on social media–based hospital service quality factors.

## Methods

### Data

To investigate the opinions about the quality factors of hospitals in South Korea, particularly the quality of pediatric hospitals, we collected user-generated content from online communities hosted by Korean Web portals. Preliminary examination showed that online communities specializing in pediatrics served as places for parents to actively discuss (through questions and answers) the quality of pediatric hospitals, sharing their thoughts, ideas, and experiences. Considering that Naver [20] and Daum [21] operate the dominant local online communities for parents and caregivers, we decided to analyze the content of these two portals.

**Figure 1:**
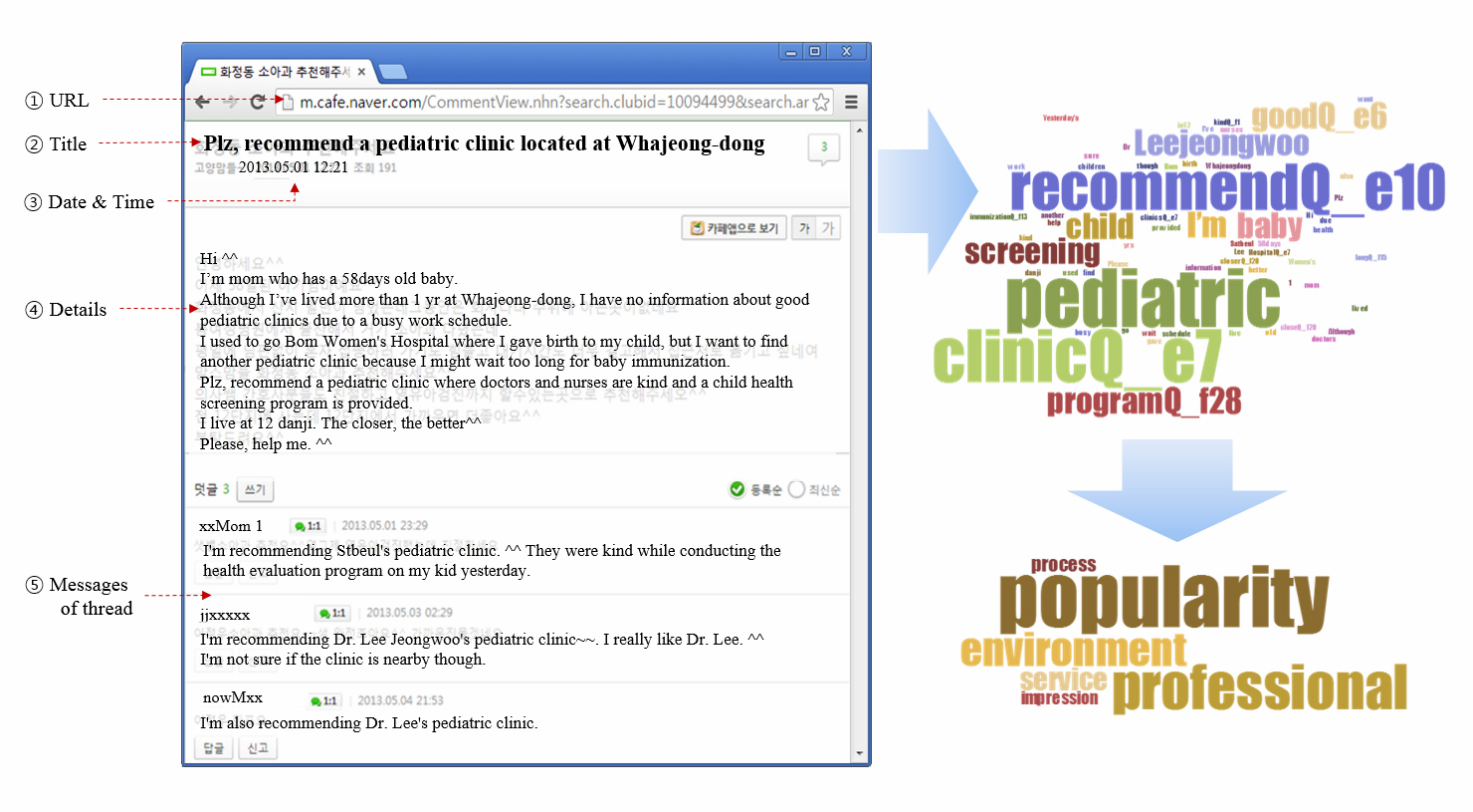
An example of online community and extracted quality factors for hospitals.

Our aim was to collect user-generated content that could contain users’ experiences with and opinions about pediatric hospitals from online health communities. The structure of the content can be divided into three levels: threads, messages, and sentences. Threads often contain several messages, whereas messages are short and often comprise only a few sentences or sentence fragments. Figure 1 is a snapshot of the hospital recommendation content of online communities, where (2) is the thread title, (4) indicates the details of the thread, and (5) contains the messages of the thread.

**Table 1:** Data statistics (from Naver and Daum Web portals).

District	Threads, n	Messages, n	Messages per thread, n	Messages containing quality factors, n
Seoul	10,832	54,392	5.02	12,421
Daegu	8072	47,419	5.87	4240
Busan	5965	28,910	4.85	9509
Daejeon	3952	22,475	5.69	2358
Incheon	775	5184	6.69	1525
Gwangju	2826	15,368	5.44	2012
Total	32,422	173,748		32,065 (18.45%)
Average	5403.66	28,958	5.59	

Table 1 shows statistics on the content from the selected parents/caregiver online communities from Naver and Daum between April 2007 and May 2013. We obtained 32,422 threads and 173,748 messages. On an average, there were 5.59 pediatric hospital-related discussions per thread among the selected regions’ community members. More specifically, 18.45% out of the total messages contain clue expressions for hospital quality factors.

**Figure 2:**
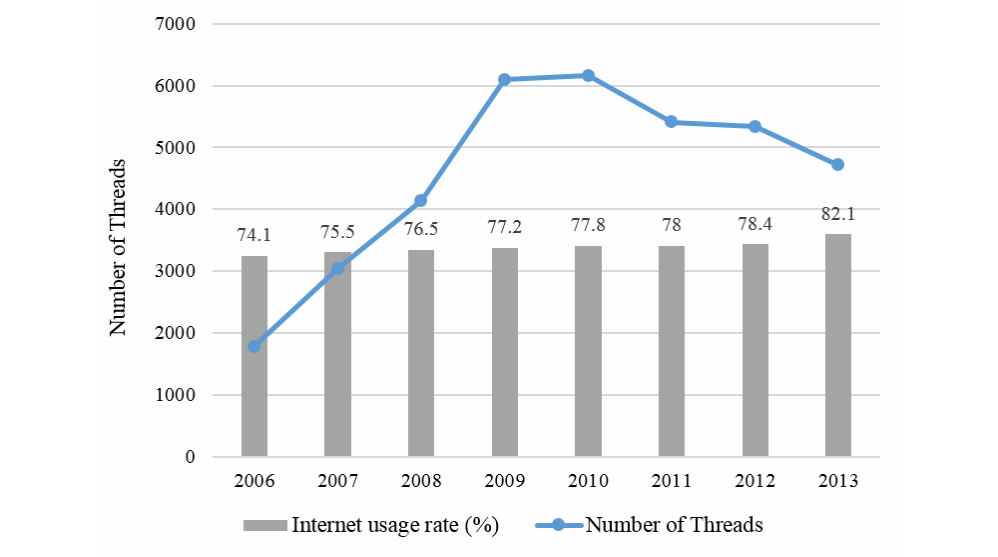
Internet usage rate and number of threads.

It is worth noting that over the sample period, there were increases in both Internet usage rate in South Korea and the content in online health communities on the two portal sites. As of 2013, the number of Internet users in South Korea was 40,080,000, (ie, 82.09% of the population) [22]. About 50% of Internet users accessed social media for personal use, and more than 70% of users have used an online community between 2010 and 2012. In the case of user-related content in online health communities, the number of threads has rapidly increased since its launch in 2003 but the number seems to have stabilized since 2010. In Figure 2, we present both these trends; our proposed method will properly address such changes in volume (the number of threads for 2013 was extrapolated using data available up to May 2013).

### Derivation of Social Media–Based Key Quality Factors for Hospitals

To efficiently manage social media data in terms of hospital service quality measurement, we defined social media-based hospital service quality factors by referring to previous quality models. However, it is also worth noting that there exist several important research studies [23-29] on service quality models for hospitals. In terms of measuring patient satisfaction, the SERVQUAL model [23], which consists of five dimensions: (1) responsiveness, (2) reliability, (3) assurance, (4) tangibles, and (5) empathy, is the most widely used tool [24]. The SERVQUAL model helps obtain customer ratings of perception and expectation on an ordinal scale. Due to its innate incompleteness, several approaches employ a modified SERVQUAL approach. For example, [25] added two quality dimensions, “caring” and “patient outcomes”, to SERVQUAL, and [26] added “core medical outcomes” and “professionalism/skill/competence”.

In terms of consumer perspectives, five quality factors for health care providers can be defined: (1) warmth, caring, and concern, (2) medical staff, (3) technology and equipment, (4) specialization and scope of services available, and (5) outcome [27].

For use in the hospital accreditation process, the Joint Commission [28] defined nine quality dimensions for hospitals: (1) efficacy, (2) appropriateness, (3) efficiency, (4) respect and caring, (5) safety, (6) continuity, (7) effectiveness, (8) timeliness, and (9) availability. The Joint Commission model is more comprehensive because it encompasses SERVQUAL and the five quality factors for health care providers.

In an effort to develop a valid and reliable instrument for hospital management’s strategic and operational decision-making, the Key Quality Characteristics Assessment for Hospitals model was developed [29], which consists of eight factors: (1) respect and caring, (2) effectiveness and continuity, (3) appropriateness, (4) information, (5) efficiency, (6) meals, (7) first impressions, and (8) staff diversity. The model used inputs from both health care providers and consumers. However, the existing service quality measurement models are not appropriate for interpreting social media data in terms of a hospital’s service quality because users write their experiences without predefined formats or principles.

**Figure 3:**
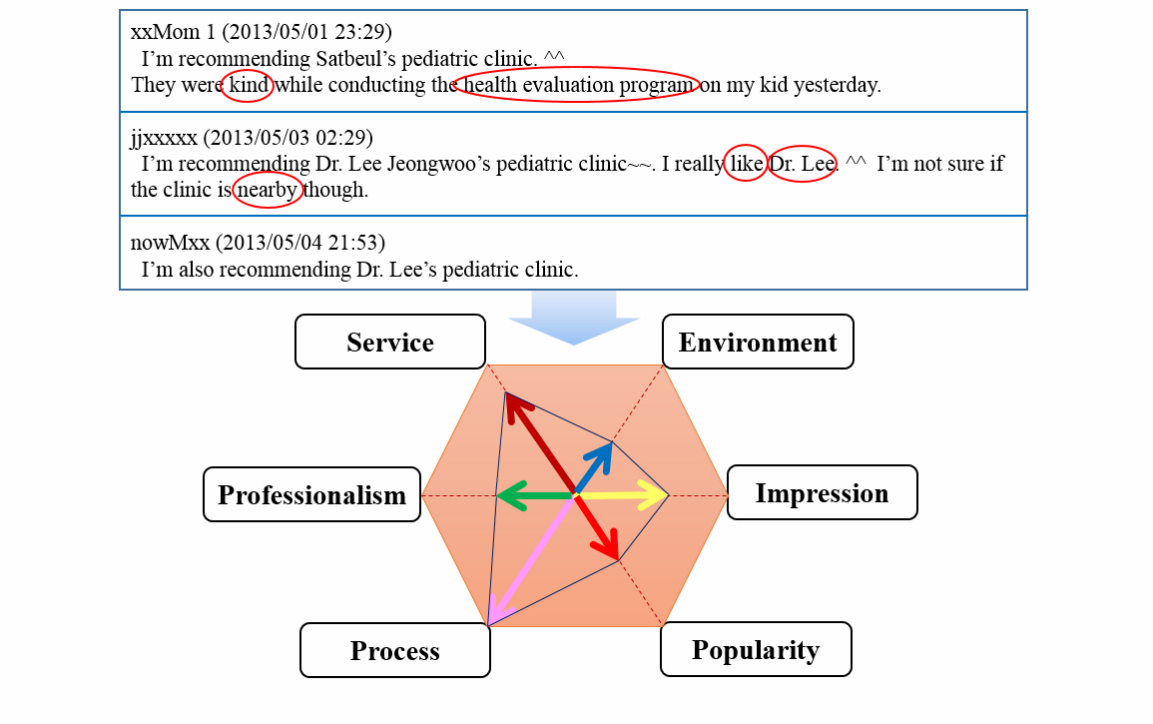
Social media-based key quality factors for hospitals.

To this end, we manually listed all the keywords that appeared more than five times in our corpus. Then we selected only terms related to services quality, for example terms regarding the status, impression, and treatments of hospitals. We finally resolved the model for social media–based hospital service quality factors to consist of six quality factors (ie, “service”, “professionalism”, “process”, “environment”, “impression”, and “popularity”), as shown in Figure 3. Each service quality factor has an average of 1000 keywords as features that can be used for analyzing the content of user-written messages. The six service quality factors have their own subcategories and subordinating items, as listed in Table 2. Each quality factor has more than one subcategory, numbered from f1–f29 and e1–e10, according to their functional or emotional characteristic, respectively.

**Table 2:** Detailed categorizations and subordinate items of social media–based hospital service quality factors.

Quality factor	Detailed quality factor	Description
Service	Kindness (f1/f2)	Kindness, courtesy related to doctor/nurse/hospital staff manners
		Diagnose and explain in easy words/rough detail
	Faithfulness (f3/f4)	Response to a request faithfully/carefully
Professionalism	Professionalism (f5/f6/f7)	Technical knowledge, skills
		Special medical courses, professional
		Professor, director (rank)
	Skill (f8/f9/f10)	Medical procedures/skills, experience
		Side effects, complications, medical malpractice, safety
		Treatment effects, speed
	Treatment (f11/f12/f13/f14)	Hospitalization, outpatient, inpatient
		Accuracy
		Antibiotics, injections, prescription drugs (powder, liquid medicine, cold medicine)
		Diagnosis, treatment, prescription, cure, appropriate (or over-) treatment
Process	Speed (f15/f16)	(Short/long) waiting time, dose standby, receiving the relevant treatment
		Responding immediately to changes, ability to cope with emergencies
	Cost (f17)	Low (or reasonable)/expensive medical costs
	System (f18/f19)	Efficient business processes (reception and express services), questionnaires, (basic/optional) medical care
Environment	Convenience (f20/f21/f22/f23)	Transportation, distance (from residence), parking facilities
		Reservations
		Waiting room, convenient facilities
		Office hours, dates, evening hours (weekends, Sundays, and late hours), closed hours
	Sanitation (f24/f25)	Cleaning, management
		Disposable products, sanitary ware
	Facility/ Equipment (f26/f27/f28/f29)	Hospitalization
		CT, MRI, equipment, tools, operating room, doctor’s office
		Surgery, physical therapy, various tests, therapies, health screenings, hospital rounds
		Hospital size
		Types of hospital (public health center, university hospital, hospitals, clinics, and private hospitals)
Impression (e1/e2/e3/e4/e5)		Image of the hospital, atmosphere
		Reliability, favorite hospitals, physician
		Signs, new doctors, or hospital encountered for the first time
		Tired of existing hospital
		Impression of the doctor, doctor’s information (ie, mood, personality, gender, age)
Popularity (e6/e7/e8/e9/e10)		Rumor, tradition, reputation
		Hospital name
		Media, advertising
		Well-known doctor (doctor’s name)
		Anyone who knows the hospital

### Text Mining for Hospital Quality Factors Extraction and Recommendation Classification

#### Overview

**Figure 4:**
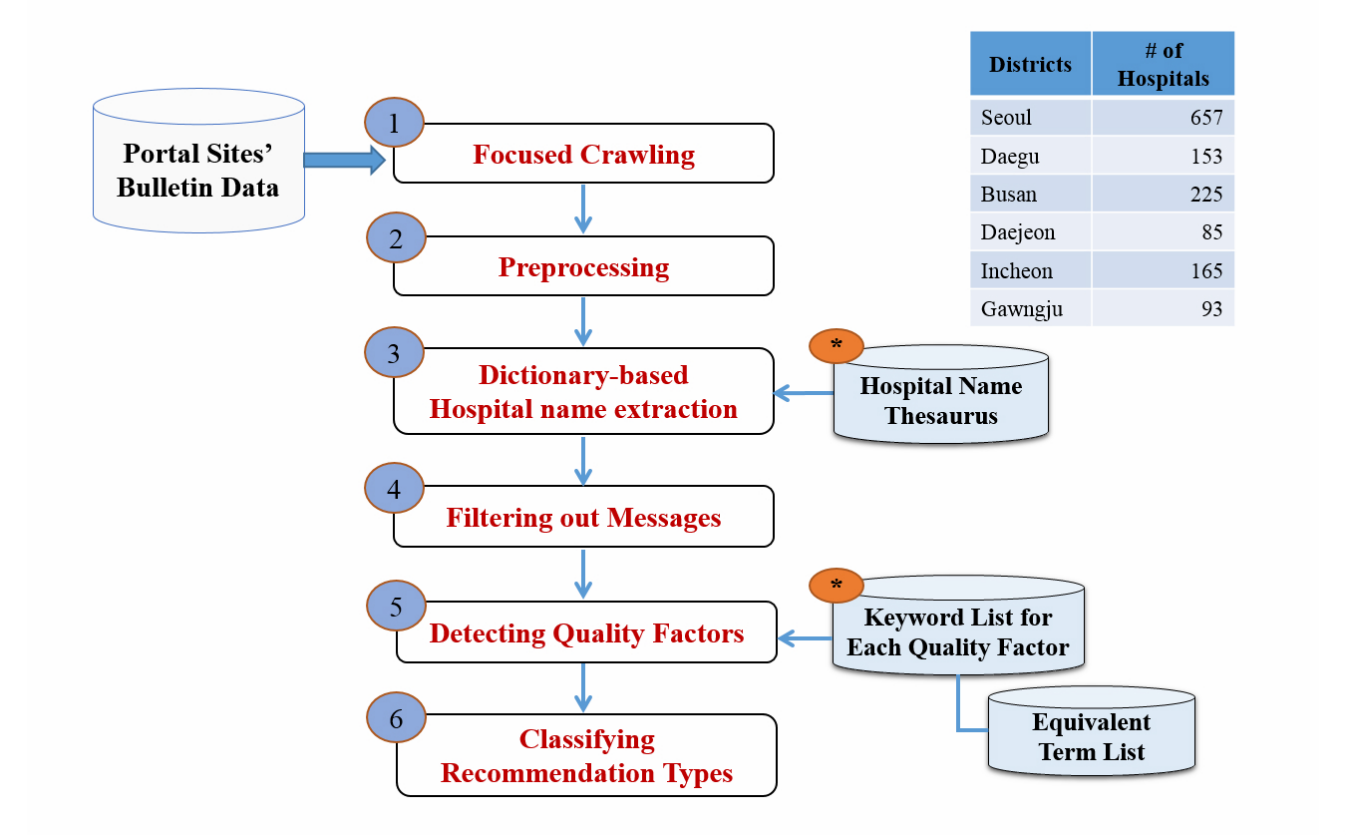
Steps for detecting quality factors and recommendation classification.

We implemented a set of text mining modules for our social media–based hospital service quality factors model-based extraction and classification. The process consists of six steps as shown in Figure 4: data preparation through crawling, data preprocessing, hospital name extraction, message filtering, quality factor detection, and recommendation type classification. After data aggregation via focused crawling, the HTML elements are removed. For every message, the next four natural language processing steps are applied to detect quality factors in the messages and to compute the hospitals’ recommendation type based on them. Although our approach can be applied to various types of domains in online health communities, we targeted messages for analysis that explicitly mentioned pediatric hospitals/clinics and discussed the quality factors related to those hospitals/clinics.

#### Step 1. Focused Crawling

The two Web portals, Naver and Daum, operate various kinds of online communities arranged by region and subject. For example, the Naver portal has approximately two million regional communities. We note that each region includes at least one parent community with active members, high popularity, and vigorous online activity. We opted for these local online communities as our text mining information sources. Each online community provides a search function that allows us to identify particular webpages with “pediatric” and “recommendation” keywords. The webpages from the selected online parent communities were crawled and stored in HTML format.

#### Step 2. Preprocessing

HTML tags and non-textual information such as images, JavaScript codes, and advertisements were deleted from the extracted files. For effective handling of hypertext markup language (HTML) content, we used the Beautiful Soup library, designed in the Python programming language [30].

#### Step 3. Dictionary-Based Hospital Name Extraction

Often, when people mention hospitals in a social context in Korean, they use a range of hospital names, mostly expressed through acronyms or abbreviations. To counter this problem, we built a hospital name dictionary covering the pediatric hospitals in the six big cities (ie, Seoul, Daegu, Busan, Daejeon, Gwangju, and Incheon) in South Korea. The hospital names were extracted from the Health Insurance Review and Assessment Service website [31]. This dictionary is a value (expression) mapping table that adds acronyms or abbreviations for every hospital name observed in user-written sentences in the online communities. Using this dictionary, our text mining module performs a specially designed stepwise expression normalization procedure, based on the textual edit-distance similarity [32] between the synonyms in the dictionary and the expressions in the sentences. This was used to find canonical hospital names for use in raw hospital representations.

#### Step 4. Filtering Out Messages

Once the canonical hospital names were extracted from the messages, we filtered out messages in which hospital names were not mentioned or which were ambiguous, so as to consider only messages that mentioned hospital names clearly. In addition, we selected candidate messages that recommended or in some other way mentioned hospitals.

#### Step 5. Detecting Quality Factors

This is the main step for identifying textual representations of quality factors for hospitals. By utilizing the keyword lists (ie, a dictionary of quality factors), we detected and extracted the service quality factors from each message. The techniques used in this task are derived from dictionary-based named entity recognition [33]. If service quality factors are identified, we count the number of occurrences for further analysis. In addition, to deal with known difficulties of Korean language processing, we used an equivalent term list derived from high frequency terms observed in our data collection.

#### Step 6. Classifying Recommendation Types

Based on the expressions of identified quality factors, this step classifies the recommendation type of each message, that is, whether a hospital is recommended or not. As a simple implementation of keyword-based sentiment analysis, it counts the number of sentimental keywords for and against recommendation [9]. To consider possible changes of type within sentences in a single message, we use a modified version of mood flow analysis [34]. As results, the classified types are positive (ie, recommended), neutral, bilateral, and negative (ie, not recommended). If we simply summarize the classification results, 35.86% (47,046/131,191) are hospital recommendations where 131,191 is the total number of quality factors considered as one of four classified types, and 47,046 is the number of quality factors showing positiveness. It is to be noted that a single message can have more than one qualify factor. However, we reduced the classification categories to two types (ie, positive or negative) by viewing it as a binary classification problem; misclassification of outcome type is a serious concern, particularly when two different polarities coexist in the same message.

### Manual Evaluations

Before analyzing in depth the results of Steps 5 and 6 in various ways, we performed manual evaluations on the extraction and classification results for things such as hospital names, quality factors, and recommendation types. For the evaluation of hospital name extraction, we performed human tagging of a random sample of 9450 messages, which amounted to 5.4% of the total messages. In addition, for the evaluation of quality factor detection and recommendation type classification, we selected the five most frequently occurring hospitals/clinics from the six regions and manually checked their subordinating quality factors and recommendation type to check

if their extractions/classifications were correct. The evaluation results are summarized in Table 3.

**Table 3:** Evaluation results (reported as percentages).

Region	Evaluation category						
	Hospital name extraction			Quality factors detection			Recommendation type classification
	Precision	Recall	F1	Precision	Recall	F1	Precision
Seoul	68	83	75	93	95	94	76
Daejeon	91	72	80	96	91	93	82
Daegu	86	65	74	97	80	88	70
Gwangju	91	66	77	96	83	89	64
Incheon	79	77	78	88	93	90	61
Busan	87	73	79	98	82	89	67
Average	84	73	78	95	87	91	78

### Hospital Name Extraction

We achieved a recall (the proportion of hospital names in the original text that were extracted correctly) of 73% and precision (the proportion of extracted hospital names that were correct) of 84%, giving F1 = 2 × (precision) × (recall)/(precision + recall) = 77.7%. The measure F1 is the harmonic mean of precision and recall. Although accuracy measures of 80%-90% have often been achieved in named entity extraction in English, there are very few cases of such high accuracy in informal texts written in Korean due to various types of acronyms, misspellings, and frequently incorrect spacing between words. These factors are currently performance bottlenecks together with the fact that Korean is an agglutinative language.

### Detecting Quality Factors

Quality factor detection is a kind of keyword matching that checks if a given word exists in one of the categories of the six quality factors. Its performance is slightly over 90% due to errors in word spacing and homonym problems of the Korean language. The currently achieved F1 score is considerably satisfactory. However, the detection performance could be enhanced further by considering compound nouns and more sophisticated processing of erroneously written sentences for clearer word spacing.

### Recommendation Classification Using Sentiment Analysis

Our recommendation classification, a type of sentiment analysis, is a challenging task in that it should track polarity transitions within a message. In spite of handling polarity transitions within a message by referring to [34], the verified classification performance is 77.8% on average. This indicates that sentiment analysis in Korean still has room for further improvement. Nevertheless, our quality factor detection and sentiment analysis provides the option to facilitate the in-depth analysis of messages by region.

## Results

### Overview

By applying the text mining techniques introduced earlier to online health community messages, we obtained a collective intelligence driven by our social media–based hospital service quality factors model. As a first step to investigate the potential of this knowledge, we analyze the occurrence patterns of preferred quality factors among different big cities in South Korea.

### Overall Social Media–Based Hospital Service Quality Factors Distributions by Region and Time

Previously, labor-intensive surveys have been used for hospital service quality evaluation. In contrast with such costly manual surveys, we can easily access multidimensional analysis results from the occurrence ratios of keywords included in one of the six quality factor categories. The occurrence ratios of each quality factor signify caregivers’ preferences when they select pediatric hospitals/clinics.

**Figure 5:**
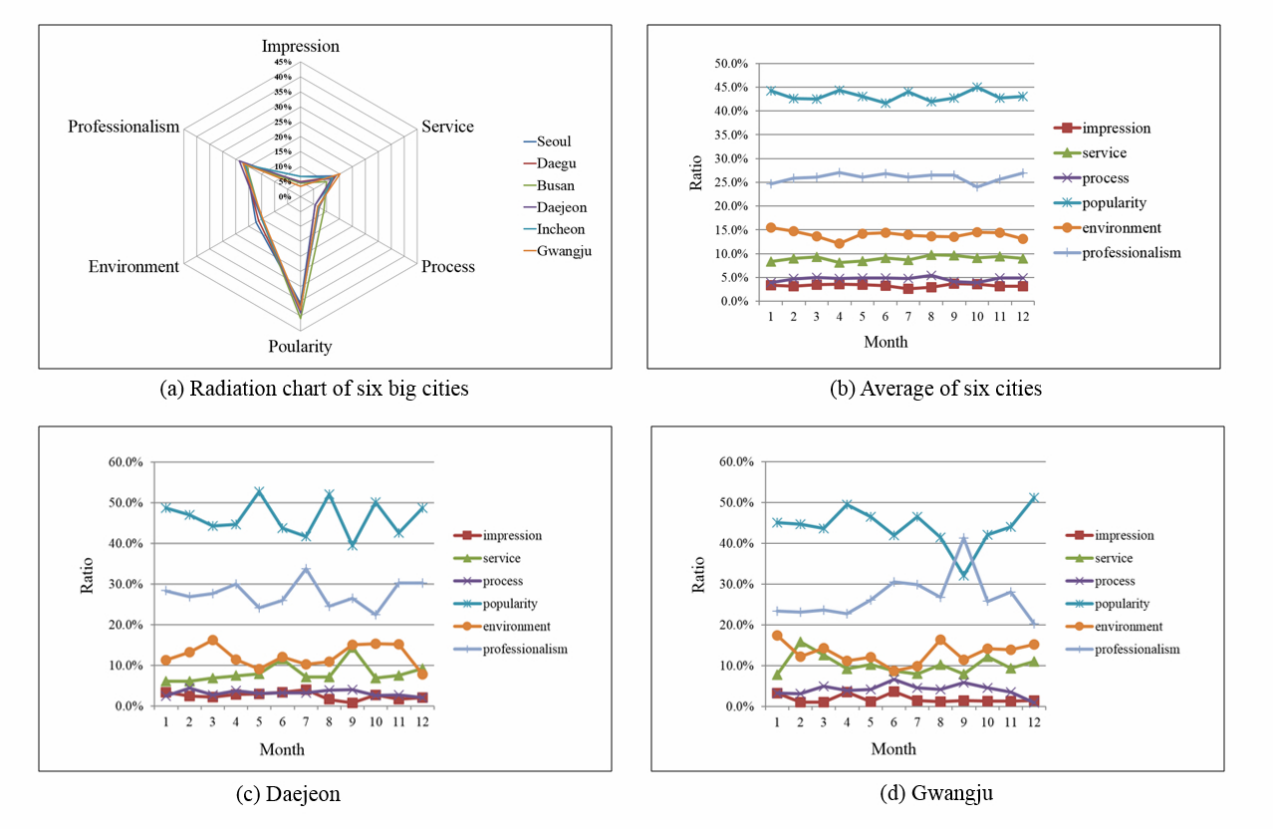
An example of overall quality factor distributions.

Panel (a) of Figure 5 gives a high-level view of the six key quality factor distributions for the six largest cities (ie, Seoul, Daegu, Busan, Daejeon, Incheon, and Gwangju) in South Korea. The plots in panel (a) of Figure 5 indicate the occupying ratios of each quality factor for each region. Although there are small variations among the different six quality factors, two distinctive factors (popularity and professionalism) occur most frequently.

Such trends are maintained every month, as shown in panel (b) of Figure 5, where the raw frequencies of quality factors are averaged over all regions. In contrast, when we observe regional data, the mean relative frequency ratios of their constituting factors differ somewhat by region and season. Panels (c) and (d) of Figure 5 show the mean relative frequency ratios per month during 2006-2013 for two selected cities, Daejeon and Gwangju, respectively. The numbers in panels (b)-(d) of Figure 5 are given as percentages of occurrence.

We assume that the analysis results driven by social media–based hospital service quality factors form a kind of collective intelligence that represents caregiver beliefs regarding the quality of local hospitals and can be effectively used to discover hospital recommendations within online health communities. Our analysis provides insight into regional and seasonal trends in quality factors that can be obtained from observing pertinent keywords/terms.

### Case Study of Negative Attitudes

#### Overview

Based on the firm belief that there exist clear differences in the distributions of social media–based hospital service quality factors according to recommendation types, we disaggregated the negative parts from the previous results to reveal hidden regional relationships between the factors. For this, we used sentiment analysis that decomposes user-written messages into two types (ie, recommended and not recommended).

**Figure 6:**
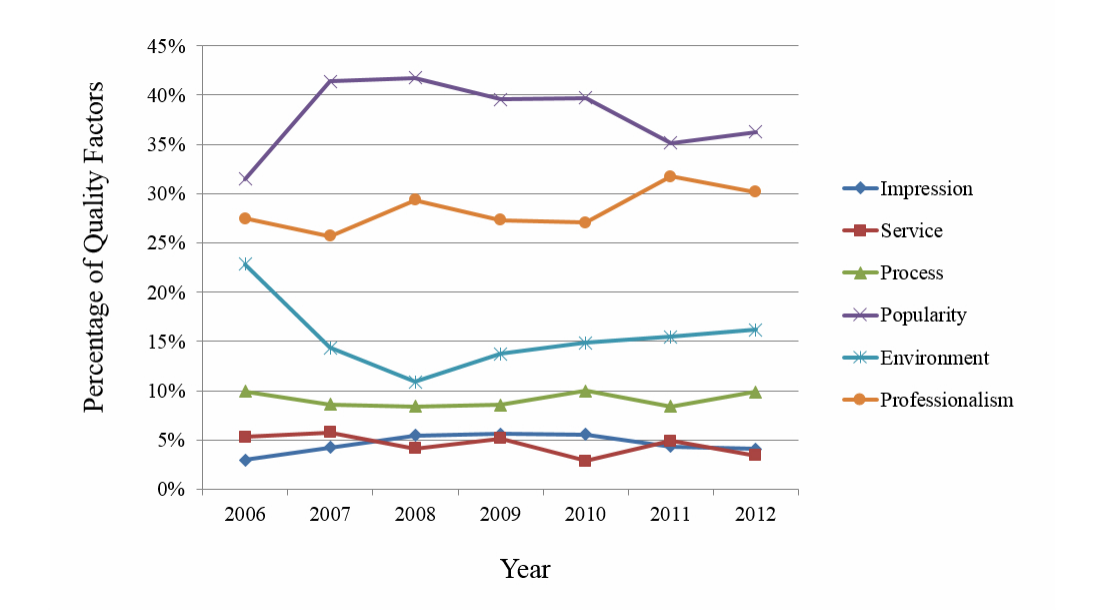
Negative attitude trends by year (for six cities).

A large number of users may have positive or neutral attitudes about their hospital experiences, even though one certain user had a negative attitude. Based on our observations, on average, 8% of the samples were identified as negative cases if we include conflicting cases (where positive and negative sentiment coexist in a single message) as negative ones. Figure 6 shows the percentages of negative comments on social media–based hospital service quality factors per year during 2006-2013 for the six major cities of South Korea. We could say that it shows the domestic characteristics of a negative attitude towards pediatric hospitals.

#### Influenza Pandemic in 2009

We draw this conclusion from the influenza pandemic in 2009. Not only South Korea but the world suffered from a new influenza A in 2009 [35]. The most severe period for South Korea was approximately from October to November of that year; the effect of the pandemic slowly reduced after December 2009. We verified 78% of influenza-related posts collected from online health communities that appeared in 2009. The details of posts mostly consisted of questions and answers, about which hospital had vaccines to spare, or how long it would take to see a doctor and get medicine prescribed. In reality, the supply of influenza vaccine was considerably insufficient, and it is clear that most patients had trouble finding hospitals to immunize their children. As people streamed into local hospitals, they posted complaints about long waiting times. In other words, health care quality received a poor grade for that period.

Figure 6 shows caregivers’ strong negative responses in 2009. Since there was also an increase in the volume of user-generated content in 2009 as seen in Figure 2, we used the percentage of quality factors instead of the mere frequency. In fact, although the occurrence of negative content regarding all six quality factors increased, we observed that the relative shares of “popularity” and “professionalism”, the top and second major factors, significantly decreased in 2009. Interestingly, we observed increases in the relative shares of “process” and “environment” in 2009, the sum of which also accounts for a significant proportion of frequencies. This implies that the experience of parents and caregivers in pediatric hospitals was often poor and their dissatisfaction increased, especially with regard to inefficient treatment process such as long waiting times or poor experience with reservation. To observe social media–based hospital service quality factors more closely, we looked at the interrelated quality factors in terms of the long waiting times at hospitals, such as process and environment, as shown in Table 4.

For the “process” factor, the top three most frequently occurring items were “long term”, “right now”, and “hospitalized” at 73%, 11%, and 10%, respectively. For the “environment” factor, the different types of keywords that characterized each corresponding factor revealed the public’s discontent and complaints about hospital services.

**Table 4:** In-depth analysis of negative attitude in 2009.

Process		Environment	
Items	Share, %	Items	Share, %
Long term	73	Reservation	48
Right now	11	Shot	13
Hospitalized	10	Examination	9
Basic	5	Health care center	7
Other	1	Other	23

#### Emergency Management in Local Cities

**Figure 7:**
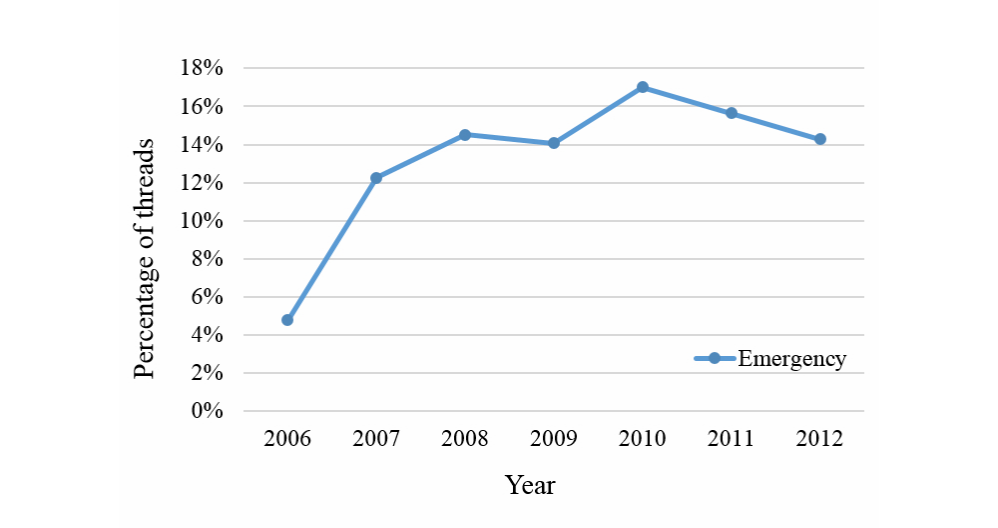
Percentage of threads that mention “Emergency” in the Daegu region.

Patients and caregivers occasionally visit general hospitals for emergency care. As can be seen in Figure 7, we observe that the “emergency”-related threads in the Daegu region increased steadily and continuously over several years until 2010. Presuming that they represent an emergent event posted in online communities, we note that approximately 5% of the threads talk about whether particular hospitals provide prompt and satisfactory emergency medical services.

In many cases, general hospitals are expected to have sufficient capacity to deal with an emergency. For example, a person who has an injured child desperately seeks hospitals open at midnight. Such people may think that the first general hospital able to provide care has competent medical staff and the necessary emergency care equipment. However, the reality in local cities is often not satisfactory. Table 5 shows that emergency-related terms often co-occur with five local general hospital names in many negatively opinionated threads (and are classified as not recommended). In this regard, we become familiar with which quality factors need to improve by analyzing social media–based hospital service quality factors. For example, “process” and “environment” factors are often detected in threads related to emergency.

**Table 5:** Co-occurrence ratios in negatively opinionated threads (Daegu region).

Co-occurrence pair	Percentage
Emergency & Hospital D1	22
Emergency & Hospital D2	16
Emergency & Hospital D3	7
Emergency & Hospital D4	31
Emergency & Hospital D5	8

One of the key reasons for this phenomenon is the small number of pediatric registrars in local general hospitals. The problem is endemic: applicants to the pediatric residency programs of local general hospitals in Daegu are lacking. Only the top two general hospitals met the required enrollment numbers in 2011. The problem can lead to poor quality of medical services, including first aid and emergency treatment. Some journalists have pointed out the need to improve hospital capacity, capability, and preparedness in the event of an emergency. The other reason is that hospital information in the local area is not well circulated. People often could not find adequate hospitals in an emergency, so they posted questions about which hospitals were available.

If the key quality factors for hospitals are detected and analyzed automatically and periodically, we can determine what kind of medical services are necessary to contain a highly contagious epidemic. Health authorities can implement a policy that could help patients based on immediate and tangible qualitative observations. From the point of view of a patient, they could discover which negative quality factors were prevalent among nearby hospitals, and hence find better medical services in other areas. Moreover, the analyzed patterns are meaningful advisory information for government policy makers because they explain the claims of caregivers who go to pediatric hospitals. Such information is helpful for both national and local health policy makers.

## Discussion

### Principal Findings

With the growing popularity of Web 2.0, a number of caregivers who have children are participating in reviewing, recommending, and exchanging opinions about pediatric hospitals. In this regard, a thorough understanding of the qualitative measures of a hospital could be important for selecting an appropriate pediatric hospital/clinic. Therefore, our contributions are threefold: (1) we define six types of quality factors feasible for social media–based hospital service quality analysis, (2) we propose a text mining approach to extract several hospital quality factors and to classify the messages into recommendation types (recommended or not recommended), and (3) we provide an in-depth analysis that can be used for many shareholders including caregivers, hospital staff, and government officials of health care policy.

### Limitations

Despite the value of the new analysis generated by this work, this study has several limitations in terms of data coverage. Social media, like other tools that assess public opinion, lends itself to selection bias, and it is difficult to assess whether this study population represents a fair sample of the general population [36]. In addition, owing to the limited information (accounting for regional tendencies), more in-depth analysis as to why different fluctuation patterns occur is required. However, it goes beyond the scope of this study, as the reasons are highly correlated with demographic characteristics.

Problems such as the inherent anonymity of users and the inability to authenticate content can be slightly mitigated because, in contrast to Twitter, messages from online health communities contain more detailed information originating from real personal experiences at hospitals. As the coverage of recommendation of pediatric hospitals/clinics in social media is not without its pitfalls, the findings highlight the potential for social media to perpetuate and perhaps even promote sources of information of unknown quality.

This study is based on our firm belief that social media can be used to gauge how patients and caregivers respond to a particular hospital. However, it has been previously found that social media also has risks. Broader scope and easily accessible platforms can lead to the wide dissemination of misinformation. We may overestimate the effect of our quality factor metrics because of the misinterpretation or over-simplification of findings from other socio-scientific studies. Nevertheless, our experimental analysis shows the great potential for social media–based key quality factor analysis for hospitals.

### Next Steps

A natural next step would be to validate the results of our analysis by comparing hospital service quality as measured through the Internet user-related data with traditional quality measures as well as patient satisfaction surveys. Previously in the literature, Greaves et al [5] did sentiment analysis on online free-text comments about hospitals and compared the results with the paper-based national inpatient survey results for all 161 acute hospitals with adult services in the United Kingdom. They found that the online comments are moderately associated with the survey and thus online comments can be used to assess patients’ opinion about hospital performance. While they compared hospital ranks, they did not look into the detailed categories of hospital service quality factors that we attempted in this study. In fact, to the best of our knowledge, a patient survey on hospital service quality factors has never been conducted at the national level. Currently in Korea, there exist several government-run websites that provide information on hospitals, for example the Ministry of Health and Welfare [37], Statistical Information Service of Health and Welfare [38], and Health Insurance Review and Assessment Service [31]. However, none of these accreditation bodies provide hospital evaluation information at the patient's satisfaction level, especially in terms of the six service quality factors used in this study. We searched and found academic studies detailing sentiment analysis of social network service mentioning about hospitals in Korea utilizing the SERVEQUAL model [39,40]. However, Song [39] analyzed social media comments about three hospitals only for 3 months. Although Kang and Song [40] compared their sentiment analysis results with survey data and verified that social media results corresponded with survey results, their analysis was confined to the top five hospitals in Korea. In fact, there have been few studies verifying social media content about hospital quality to date, mainly due to the lack of hospital quality surveys conducted at the national level. If a survey of hospital service quality will be conducted at the national level, it would be important and interesting to validate how well our social media-based analysis reflects the actual quality of hospitals.

Although we leave such validation work to be conducted in future studies, real-time information extracted from online sites via our proposed methodology can be still helpful to government authorities as well as information-seeking patients at both local and national levels. Recently, information from social media has been analyzed and used at the national level in the United Kingdom. In particular, the UK government recommends the use of social media and sentiment analysis for rapidly measuring hospital performance and poor care [41] and also runs the website National Health Service (NHS) Choices [42], where people can rate the quality of care received by their family practice.

### Conclusions

This work and future studies in this field are critical to inform both research and health care communities about the current status of hospital service quality and to highlight the need for careful, evidence-based analysis of user-preferable quality factors and recommendations based on them. To further explain the possible causes of hospital selection patterns, we plan to analyze the results in conjunction with demographic data.
